# Assessment of knee flexion in young children with prosthetic knee components using dynamic time warping

**DOI:** 10.3389/fresc.2023.1227870

**Published:** 2023-08-25

**Authors:** Mark D. Geil, Zahra Safaeepour

**Affiliations:** ^1^ Wellstar College of Health and Human Services, Kennesaw State University, Kennesaw, GA, United States; ^2^ Department of Human Performance and Health, University of South Carolina Upstate, Spartanburg, SC, United States

**Keywords:** gait, pediatrics, prosthetics, dynamic time warping, motion analysis

## Abstract

**Introduction:**

Analysis of human locomotion is challenged by limitations in traditional numerical and statistical methods as applied to continuous time-series data. This challenge particularly affects understanding of how close limb prostheses are to mimicking anatomical motion. This study was the first to apply a technique called Dynamic Time Warping to measure the biomimesis of prosthetic knee motion in young children and addressed the following research questions: Is a combined dynamic time warping/root mean square analysis feasible for analyzing pediatric lower limb kinematics? When provided at an earlier age than traditional protocols dictate, can children with limb loss utilize an articulating prosthetic knee in a biomimetic manner?

**Methods:**

Warp costs and amplitude differences were generated for knee flexion curves in a sample of ten children five years of age and younger: five with unilateral limb loss and five age-matched typically developing children. Separate comparisons were made for stance phase flexion and swing phase flexion via two-way ANOVAs between bilateral limbs in both groups, and between prosthetic knee vs. dominant anatomical knee in age-matched pairs between groups. Greater warp costs indicated greater temporal dissimilarities, and a follow-up root mean square assessed remaining amplitude dissimilarities. Bilateral results were assessed by age using linear regression.

**Results:**

The technique was successfully applied in this population. Young children with limb loss used a prosthetic knee biomimetically in both stance and swing, with mean warp costs of 12.7 and 3.3, respectively. In the typically developing group, knee motion became more symmetrical with age, but there was no correlation in the limb loss group. In all comparisons, warp costs were significantly greater for stance phase than swing phase. Analyses were limited by the small sample size.

**Discussion:**

This study has established that dynamic time warping with root mean square analysis can be used to compare the entirety of time-series curves generated in gait analysis. The study also provided clinically relevant insights on the development of mature knee flexion patterns during typical development, and the role of a pediatric prosthetic knee.

## 1. Introduction

Many limb prostheses are designed to be as biomimetic as possible (1, 2). Joint components seek to match the flexion and extension properties of the anatomical joints they replace. Material properties of elastic components are chosen to approximate the energy storage that occurs naturally in physiological tissues (3). In some limbs, active, motorized components attempt to replicate the energy generation that would occur in the muscles. While some design approaches seek only to match the kinetic or energetic function of the anatomical limb and not necessarily its motion, a genuinely biomimetic prosthesis would match both kinematics and kinetics.

A challenge in the analysis of human locomotion is that many important outcomes are time-series curves throughout a gait cycle. Most statistical analyses focus on some element of those curves, such as a local maximum or minimum, or the timing of events or extrema. For example, multiple articles analyzing the motion of prosthetic knee joints utilize single data points extracted from kinematic and kinetic time series (4–9). This is the first and most common of the three techniques for analysis of time-series curves suggested by Derrick and Thomas as follows: (1) identify the magnitude or timing of “pertinent discrete points”, (2) use the entire curve to calculate a subsequent variable such as an average, or (3) transform the curve for additional analysis, such as with differentiation (10). In particular, the comparison of two different time-series curves can be problematic. Motion analysis curves must often be time-normalized to 100% of a gait cycle to enable comparison. However, this step precludes any comparison of the actual duration of one cycle vs. another. Time-shifting can also occur in techniques like cross-correlation, but this can leave unmatched data points. The Pearson product-moment correlation can compare the temporal stability of two curves but is usually applied to data with a zero lag (11). Vector field testing approaches such as Statistical Parametric Mapping use the entire time series for comparison, but when applied to motion data in biomechanics, focus on time-normalized, or “registered”, data sets (12). These analyses therefore do not provide the ability to analyze temporal comparisons between trials.

Dynamic Time Warping (DTW) was proposed in 1978 as a technique to analyze speech patterns (13). DTW takes two signals and, similarly to cross-correlation, plots them each as rows and columns in a matrix for comparison. Whereas typical curve comparison is limited to determining the Euclidean distance between matched points, essentially determining differences via the diagonal of the matrix, DTW allows the alignment of a point from one series that does not correspond to the matching time point of the other series. This is especially beneficial when one series is longer than the other or when inflection points occur at different times. The method is constrained such that warping does not change the temporal order of the series. The process produces a nondimensional Warp Cost (WC), with a higher value indicating greater temporal dissimilarity between the original curves.

Thies et al. applied DTW to human motion and added a measure of root mean square error between the amplitudes of the two curves post-warping (14). Thies et al. later applied the method to measure variability in the motion of upper limb prosthesis users (15). The technique has been applied more recently to cardiovascular geometries (16) and to comparisons of social gestures in individuals with autism (17). To our knowledge, the technique has not been utilized to assess lower-limb prosthesis movement or the biomimesis of a prosthetic limb.

The DTW approach is potentially valuable for assessing the effects of a relatively new prosthetic prescription protocol in young children. Historically, a working prosthetic knee component is not prescribed until the child is capable of independent standing and initial walking (18–20). A newer “Early Knee” protocol prescribes a knee in the first prosthesis, intending to limit the formation of clearance-related gait adaptations (21, 22). Previous research has been limited to qualitative description of stance phase prosthetic knee flexion and quantitative analysis of only peak swing phase prosthetic knee flexion. However, clearance adaptations are highly dependent on the overall shape of the knee flexion curve in the late stance and throughout the swing. In addition, stance phase knee flexion has not previously been studied in this population.

The purpose of this study was to conduct the first quantitative analysis of the entire knee flexion curve comparing a population of young children with limb loss to typically developing peers to understand better to what degree prosthetic knee flexion can be biomimetic in these early walkers.

## 2. Materials and methods

### 2.1. Participants

This study was a subsequent analysis of a subset of data from a more extensive study described in Geil et al. (22). That study included a convenience sample of children aged 12 months to five years in three groups: children with limb loss in a traditional prosthetic knee prescription protocol, children with limb loss in an Early Knee prosthetic prescription protocol, and age-matched typically developing children. For the current study, we analyzed the data from two of the groups: children with unilateral limb loss at or proximal to the knee who were treated using the Early Knee (EK) protocol, and age-matched typically developing (TD) children without limb loss (Table 1). We did not analyze knee motion in the third group because for young children in the traditional protocol, the prosthetic knee is either missing or is locked in full extension.

**Table 1:** Participant characteristics.

Pair	Group	Age (mo)	Sex	Height (cm)	Body Mass (kg)	Side of limb loss	Prosthetic foot	Prosthetic knee
1	EK	18	M	83.0	12.4	L	TRS Little Foot	Otto Bock 3R38
	TD	15	M	75.6	10.5			
2	EK	26	F	80.0	7.9	R	TRS Little Foot	Otto Bock 3R38
	TD	21	F	81.0	11.5			
3	EK	48	F	101.0	15.5	R	College Park Truper	Otto Bock 3R38
	TD	47	F	112.0	21.0			
4	EK	62	F	108.0	16.0	R	Trulife Child's Play	Otto Bock 3R38
	TD	67	F	104.0	19.2			
5	EK	69	M	123.0	24.0	R	College Park Truper	Total Knee Junior (1,100)
	TD	68	F	117.0	22.5			

Children in the EK group were all treated at Children's Healthcare of Atlanta and used their prostheses daily without assistive devices. An ABC board-certified pediatric prosthetist performed prosthetic fitting and alignment. Gait analyses were performed at Georgia State University for both EK and TD groups. The protocol was approved by ethics committees of Children's Healthcare of Atlanta and Georgia State University, and informed parental consent was obtained for each participant.

### 2.2. Procedure

Motion analysis was conducted using an eight-camera motion capture system at 100 Hz (Vicon, Oxford Metrics, UK). Sixteen markers were attached according to the Vicon Plug-In-Gait lower body model. Each subject walked at a self-selected walking speed along a 10 m walkway for ten trials while kinematics were recorded? Using Plug-in Gait software (Vicon Nexus 2.0, Plug-In-Gait), knee flexion-extension angles and timing of heel contact and toe-off events were exported.

### 2.3. Analysis

In this study, stance and swing phase variability metrics, including WC and RMS errors, were calculated for knee angle data series. Three trials were included for each subject. The total number of cycles varied within each trial but was typically 5–6. Knee flexion data were divided into single gait cycles (initial contact to subsequent initial contact) but not time-normalized. Stance and swing were analyzed separately as each represents a different mechanical function of a passive prosthetic knee joint. Each cycle was divided into stance and swing phases based on the heel contact and toe-off events.

WC and RMS were calculated using Matlab code by Thies (14). The algorithm performs a “trial-to-trial” comparison and time-warps one of the trials to match the other. Temporal “error” is defined as the Euclidian distance for each data point in the warped trial vs. the reference trial. According to (14), computation of this “error” for every possible pairing of data points provides an error surface with axes *t* and *t'* representing time in each trial. The path of minimum error defines optimal time warping f(*t'*). The warp cost is ultimately calculated as the error between the path of least error and a 45° line, which would represent a perfect temporal match. The analyses were performed for stance and swing of the prosthetic limb in the EK group vs. the dominant limb in the TD group.

Two separate two-way ANOVAs, one for WC and one for RMS, with alpha 0.05 were used to assess the role of phase (stance vs. swing) and type of comparison (bilateral for EK, bilateral for TD, and TD vs. EK). Linear regression was used to assess bilateral WC and RMS results in each group vs. age.

## 3. Results

Though the condition was not specifically recruited, all children in the EK group presented with proximal femoral focal deficiency. Children varied in age from 18 to 69 months (Table 1). Comparison data for both within-subject bilateral differences and between-subject age-matched pairs in both stance and swing are presented in Table 2.

**Table 2:** WC and RMS results in stance and swing for bilateral comparisons in EK and TD groups, and comparisons of EK prosthetic knee to TD dominant limb knee.

Phase	Variable	Comparison	Mean	Std Dev
Stance	WC	TD Bilateral	8.97	5.02
		EK Bilateral	13.93	7.39
		EK-TD	12.68	2.53
	RMS	TD Bilateral	3.98°	1.24°
		EK Bilateral	6.56°	3.27°
		EK-TD	4.84°	0.56°
Swing	WC	TD Bilateral	1.69	0.94
		EK Bilateral	4.82	2.60
		EK-TD	3.32	0.44
	RMS	TD Bilateral	3.87°	3.20°
		EK Bilateral	8.44°	4.45°
		EK-TD	8.72°	8.33°

**Figure 1:**
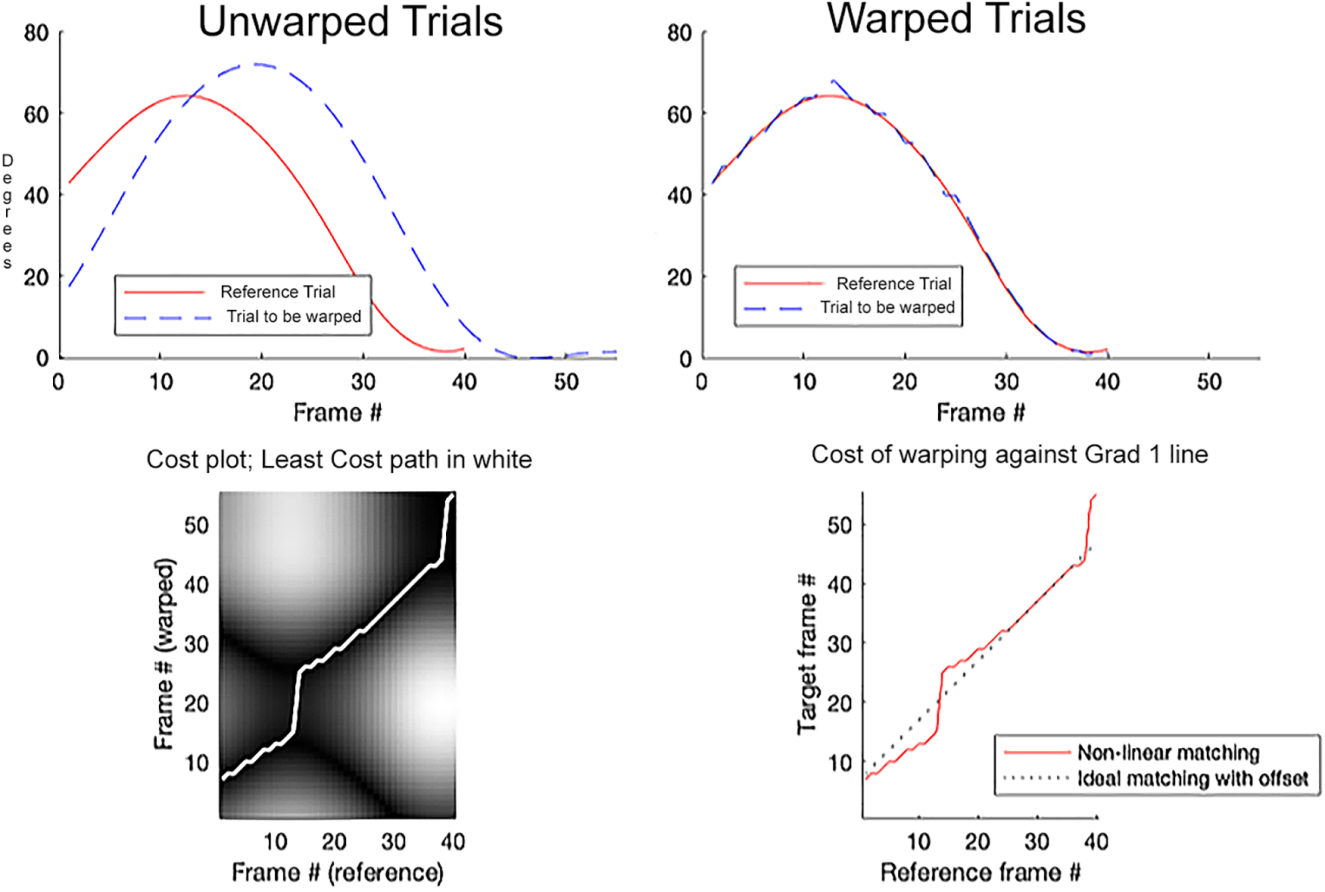
Representative sample of dynamic time warping comparing an age-matched pair from each group. Solid line represents swing phase dominant limb knee flexion for a child in the TD group. Dotted line represents swing phase prosthetic knee flexion for a child in the EK group.

The WC/RMS technique was successfully applied to knee flexion curves (Figure 1), producing a WC and RMS for each bilateral comparison and for age-matched comparisons of EK prosthetic knee flexion to TD anatomical knee flexion. Overall, WC ranged from a minimum of 0.36, comparing left to right in swing in a TD child, to a maximum of 27.17, comparing the prosthetic side to the contralateral side in stance in an EK child. RMS showed a smaller range, from a minimum of 1.21°, comparing prosthetic side to contralateral side in stance, to a maximum of 25.23° comparing an age-matched pair in swing.

Analysis of variance determined no evidence of interaction among the three groups (EK bilateral, TD bilateral, and EK-TD age-matched pairs) for either measure (WC or RMS). Therefore, main effects were examined. The WC main effect was significant (*F* = 11.58, *p *< 0.0001). WC in Stance was significantly greater than in Swing (*F* = 30.14, *p *< 0.0001), but the difference between groups was not significant (*F* = 2.31, *p *= 0.1198). The RMS main effect was not significant (*F* = 1.43, *p *= 0.2558). In both phases, both WC and RMS were lowest in bilateral comparisons within TD children.

**Figure 2:**
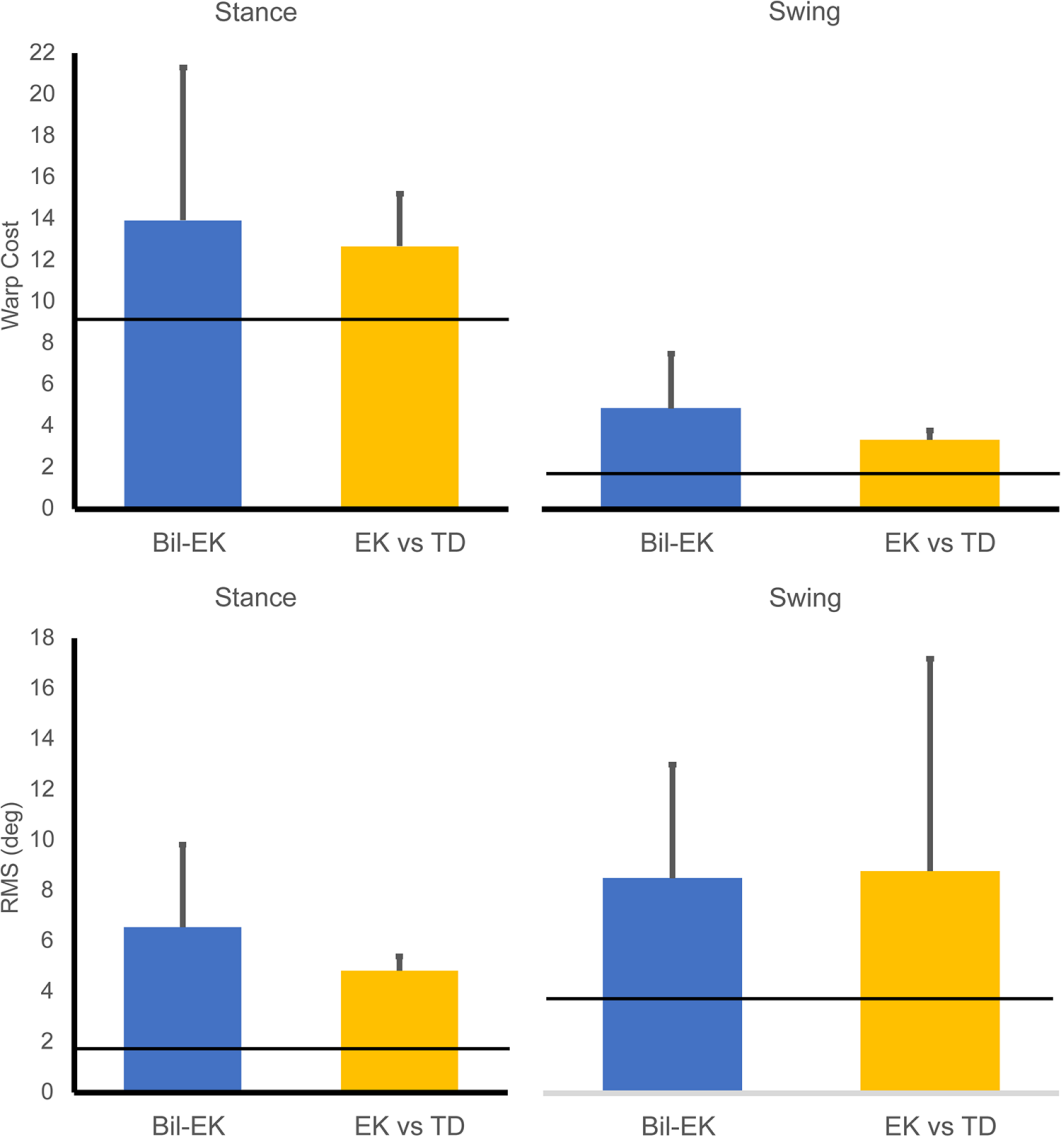
Average warp cost timing dissimilarities (top row) and root mean square amplitude dissimilarities (degrees, bottom row) in stance (left column) and swing (right column). Horizontal lines indicate the value for bilateral asymmetry in typically developing children. “Bil-EK” refers to prosthetic knee vs. contralateral anatomical knee in the EK group. “EK vs. TD” refers to prosthetic knee in the EK group compared to dominant knee in age-matched TD comparator.

For clinical interpretation, the results of bilateral comparisons for TD children were used as a baseline for comparing values in the EK group, based on an expectation of symmetry in typically developing children. On average, WC values were lower for swing than stance in both groups, while RMS values were higher (Figure 2).

In the TD group, both WC and RMS decreased with age, though correlations varied with age (Table 3). Results in the EK group were much less consistent, with weak correlations for both variables in both phases.

**Table 3:** Results of linear regressions for WC and RMS in each group vs. age.

Group	Variable	Phase	*R* ^2^	*p*
TD	WC	Stance	0.86	0.023*
		Swing	0.90	0.013*
	RMS	Stance	0.84	0.023*
		Swing	0.69	0.080
EK	WC	Stance	0.35	0.290
		Swing	0.17	0.497
	RMS	Stance	0.21	0.434
		Swing	0.02	0.800

* Statistically significant (*p* < 0.05).

## 4. Discussion

This study represents the first comparison of the complete time series of a knee flexion angle curve using dynamic time warping. The study aimed to assess if young children with limb loss can use a prosthetic knee in a biomimetic fashion if one is provided to them. Biomimetic knee use could reduce the development of gait adaptations common in this population.

Because this was a novel application of the DTW technique of Thies et al. (14), it was important to include comparisons of motion that were expected to be very similar to establish values for WC and RMS that would reflect minimal differences. Subsequent values found close to these benchmarks could then be interpreted with that knowledge. In this study, we expected minimal differences in bilateral knee motion in typically developing children. These results provided insight into typical development in this small sample. The WC was much lower in swing than stance (1.70 vs. 8.97), suggesting that the timing and curve shape of stance phase knee flexion is more variable than swing. Conversely, RMS was lower in stance vs. swing (1.84° vs. 3.87°). At this young age, even typically developing children demonstrate bilateral asymmetries in knee motion.

The study provided two ways to assess how closely prosthesis motion mimicked anatomical motion: comparison to the subject's contralateral intact limb, and comparison to an age-matched control. Both have limitations. In an individual with unilateral limb loss, the contralateral limb is not expected to represent the motion that the same individual would have without limb loss. And in young children, neuromotor development varies with age, so age-matched comparisons, while typically used, introduce variability. The results in this study were generally similar between the two types of comparison, with age-matched comparisons tending to show slightly greater biomimesis.

Regardless of comparison type, the study showed that young children with limb loss use a prosthetic knee in swing phase in a very biomimetic fashion. By contrast, the WC for bilateral asymmetry in TD children in stance, a population in which symmetry is usually assumed, was 86% higher than the swing phase WC for bilateral asymmetry in the prosthesis side vs. contralateral side in the EK population, and 170% higher than the age-matched comparisons.

In general, the DTW-RMS approach added insight over a simple comparison of peak values. For example, comparison of swing phase flexion in the 68–69 month old pair (Figure 1) showed a difference of 5.9° peak flexion, but the timing of the flexion also differed, with the TD subject reaching peak flexion 9% later in the gait cycle. In our traditional analysis, this difference in timing would have been diminished through normalization, and then not considered in a focus on the peak value. In addition, analysis of the complete curves (and not just the maxima) showed that the children with limb loss in our sample did not show age-based reductions of bilateral asymmetries in knee motion like their age-matched typically developing peers.

The study had several limitations. The small sample size does not allow the results to be generalized to the broader population of young children with limb loss. We chose to analyze stance phase and swing phase separately, because each phase has very specific implications associated with prosthetic knee hardware design. Therefore, this approach does not provide information about the feasibility of analysis of a complete cycle knee curve using DTW. In addition, analysis of individual subject results showed that the EK subject in the third age-matched pair (47–48 mo.) showed an unusually high RMS amplitude. This subject's mean peak knee flexion was 39.6° compared to a mean peak knee flexion of 71.5° for all other EK subjects. Further inspection showed that this subject had a particularly long residual limb, leaving a very short prosthetic segment distal to the knee component, which reduces the ability of a passively flexing prosthesis to flex based on inertia. When DTW and RMS are used to compare curves in a highly variable population such as this one, the individual trials should be monitored for cases when curves are substantially different for subject-specific anatomical or prosthetic reasons.

In conclusion, the study demonstrated the effectiveness of a dynamic time warping approach to analysis of time-series curves in human locomotion. The study showed that, in this small sample, all participants, including typically developing children, demonstrated high variability and asymmetry in stance phase knee flexion, while swing phase flexion of children in an early knee prosthetic prescription protocol closely matched both the contralateral limb and an age-matched typically developing child. It is important to note that we analyzed only one pair of children per age group, so inferences are limited.

## Data Availability

The datasets presented in this study can be found in online repositories. The names of the repository/repositories and accession number(s) can be found below: https://digitalcommons.kennesaw.edu/datasets/5/ Digital Commons DOI: 10.32727/27.2023/2.
